# Velocity Estimation from LiDAR Sensors Motion Distortion Effect

**DOI:** 10.3390/s23239426

**Published:** 2023-11-26

**Authors:** Lukas Haas, Arsalan Haider, Ludwig Kastner, Thomas Zeh, Tim Poguntke, Matthias Kuba, Michael Schardt, Martin Jakobi, Alexander W. Koch

**Affiliations:** 1 IFM—Institute for Driver Assistance Systems and Connected Mobility, Kempten University of Applied Sciences, Junkerstraße 1A, 87734 Benningen, Germany; 2 Institute for Measurement Systems and Sensor Technology, Technical University of Munich, Theresienstr. 90, 80333 Munich, Germany; 3 Faculty of Electrical Engineering, Kempten University of Applied Sciences, Bahnhofstraße 61, 87435 Kempten, Germany; 4 Blickfeld GmbH, Barthstr. 12, 80339 Munich, Germany

**Keywords:** LiDAR sensor, deep learning, motion distortion effect, point cloud, advanced driver assistance systems, highly automated driving, velocity estimation

## Abstract

Many modern automated vehicle sensor systems use light detection and ranging (LiDAR) sensors. The prevailing technology is scanning LiDAR, where a collimated laser beam illuminates objects sequentially point-by-point to capture 3D range data. In current systems, the point clouds from the LiDAR sensors are mainly used for object detection. To estimate the velocity of an object of interest (OoI) in the point cloud, the tracking of the object or sensor data fusion is needed. Scanning LiDAR sensors show the motion distortion effect, which occurs when objects have a relative velocity to the sensor. Often, this effect is filtered, by using sensor data fusion, to use an undistorted point cloud for object detection. In this study, we developed a method using an artificial neural network to estimate an object’s velocity and direction of motion in the sensor’s field of view (FoV) based on the motion distortion effect without any sensor data fusion. This network was trained and evaluated with a synthetic dataset featuring the motion distortion effect. With the method presented in this paper, one can estimate the velocity and direction of an OoI that moves independently from the sensor from a single point cloud using only one single sensor. The method achieves a root mean squared error (RMSE) of 0.1187 m s^−1^ and a two-sigma confidence interval of [−0.0008 m s^−1^, 0.0017 m s^−1^] for the axis-wise estimation of an object’s relative velocity, and an RMSE of 0.0815 m s^−1^ and a two-sigma confidence interval of [0.0138 m s^−1^, 0.0170 m s^−1^] for the estimation of the resultant velocity. The extracted velocity information (4D-LiDAR) is available for motion prediction and object tracking and can lead to more reliable velocity data due to more redundancy for sensor data fusion.

## 1. Introduction

Sensor data form the basis for environmental perception and, thus, the decision-making basis of autonomous systems. Today, light detecting and ranging (LiDAR) sensors are used in many autonomous systems, such as autonomous driving (AD) and advanced driver assistance systems (ADAS) [1], and building automation through people or people flow detection [2].

In LiDAR sensors, two different principles are used to measure the distance of a point to the sensor: the time-of-flight (ToF) principle and the frequency modulated continuous wave (FMCW) principle [3]. Currently, the majority of LiDAR sensors use the ToF principle. In contrast to FMCW LiDAR sensors or RADAR sensors, ToF LiDAR sensors cannot provide velocity information about OoIs within one frame.

One exemplary use case for a velocity measurement is modern vehicles’ active cruise control systems (ACC). The highly automated driving function of the ACC needs the velocity and position of the vehicle of interest (VoI) in front of the car to control the velocity of the car [4].

**Figure 1:**
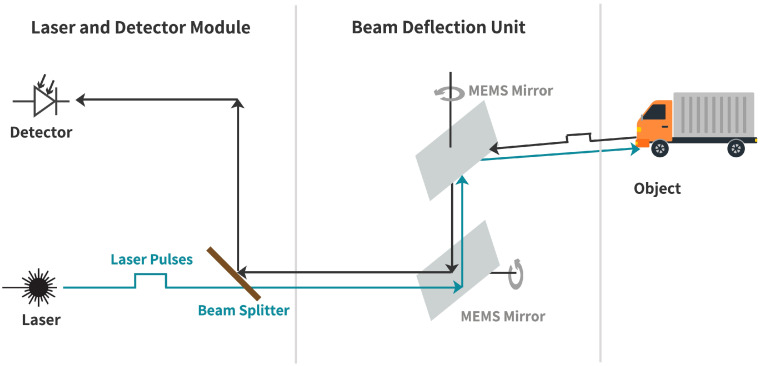
Functional principle of a scanning LiDAR sensor using two 1D microelectromechanical system based mirrors.

To perceive the environment, the sensors emit light pulses with a specific wavelength, typically ~905 nm or ~1550 nm. The emitted laser pulses are partly reflected by the object surfaces in the sensor’s FoV and detected by the LiDAR sensor. The LiDAR sensor measures the propagation time τ between the emission and detection of a laser pulse to calculate the distance *d* via the speed of light *c*, as in Equation (1). The working principle of a ToF LiDAR sensor is shown in Figure 1.

(1) $$d=\frac{c\;\cdot \;\tau }{2}$$

LiDAR sensors can be further categorized as scanning and flash LiDAR sensors. Scanning LiDAR sensors steer the laser beam pulses in the FoV to obtain the 3D view of the vehicle’s surroundings in a specific frame duration. Scanning LiDAR sensors can measure up to 200 m distance with a horizontal and vertical resolution of 0.1${}^{\circ }$ [5,6]. In contrast, flash LiDAR sensors illuminate their complete FoV at once and can measure a distance of up to 50 m due to the limited transmit power of the laser because of eye safety regulations [7,8,9]. Because of the larger measuring distance, scanning LiDAR sensors are used in different automotive ADAS and AD applications—for example, for lane departure warnings (LDW), simultaneous localization and mapping (SLAM), forward collision warning (FCW), and blind spot detection and adaptive cruise control (ACC) [9].

For many ADAS functions, not only information about the position and orientation of objects near the vehicle is essential, but also their velocity [9]. The velocity information can only be obtained using computationally expensive object tracking methods or radar sensors for the measurement of the radial velocity. Since the sensor data form the basis for the decisions of the ADAS or AD functions, these should be as reliable as possible. To react quickly to events, the information should be available as soon as possible. Furthermore, the information should be collected with different sensors and made available to the sensor data fusion module to achieve this. This is difficult to implement with conventional object tracking methods, as these require several consecutive frames to determine the velocity of an OoI. Therefore, in this paper, we present a method for the velocity estimation of objects based on the point clouds of scanning LiDAR sensors.

The paper is structured as follows. First, the motion distortion effect is generally discussed. Then, we explain the sensor specifics in more detail for the sensor used in this work. After this, the current state of the art is described. The fourth section discusses the creation of the dataset for neural network training. After the dataset, the neural network and its optimization and validation are described. Subsequently, the results are summarized, and an outlook on future applications and research is given.

## 2. Motion Distortion Effect

Scanning LiDAR sensors use the ToF principle to create a point cloud of their environment. The position of the detected point in the point cloud can be calculated from the azimuth and elevation angle with which the laser pulse was shot in the environment and the duration between sending out the pulse and detecting the reflection of the pulse. To scan the entire FoV with a defined angular resolution, the sensor has to shoot multiple laser pulses one after another with different angles in the environment. The direction in which the laser pulses are shot in the environment and the sequence in which the laser pulses succeed each other is called the scan pattern. All detected points of one scanning sequence are stored together in one LiDAR frame. The pattern used to scan the FoV of a current LiDAR sensor stays the same for all the frames measured. Because of the scanning principle of the LiDAR sensor, it takes a specific duration to scan one entire LiDAR frame.

**Figure 2:**
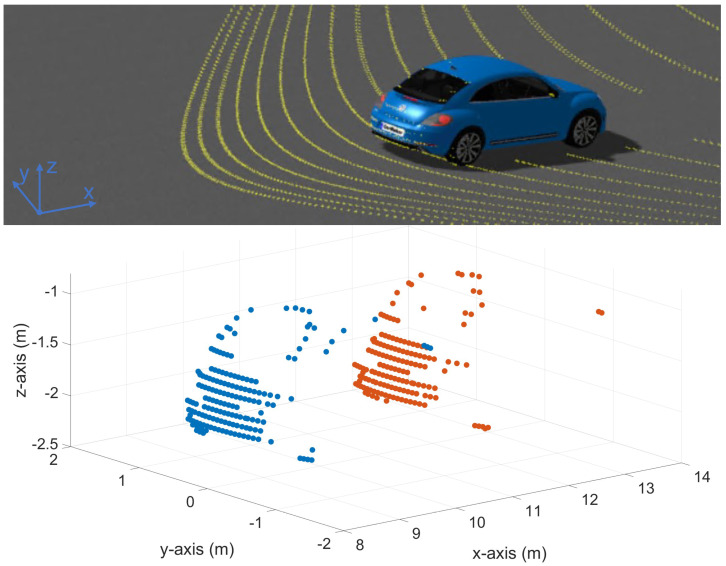
Distorted point cloud section of a vehicle with a view to the VoI’s trunk driving away from the ego vehicle with 32.77 m s^−1^ at a distance of 9 m. The second shape of the trunk, shown in red, appears due to the scan pattern of the LiDAR sensor and the position and dimension of the OoI in the FoV. This can cause a false positive detection.

Scanning an object with a relative velocity to the sensor, the object changes the position in the FoV of the sensor within one LiDAR frame. Because of this, the object is distorted due to the relative velocity between the sensor and the object. This effect is called the motion distortion effect; some authors use the term motion blur [10]. Figure 2 shows an example of an OoI in a distorted point cloud based on the motion distortion effect.

LiDAR sensors can be classified not only based on their measurement principles. Based on the different scan patterns of scanning LiDAR sensors, the sensors can be further classified into rotating and oscillating LiDAR sensors. Rotating scanning LiDAR sensors use a mechanical setup to rotate the actual scanner horizontally in the FoV. In contrast, oscillating sensors such as microelectromechanical system (MEMS)-based LiDAR sensors can be designed without mechanically rotating components [11]. The motion distortion effect affects all scanning LiDAR sensors regardless of their scan pattern. Yan et al. [11] show that the influence of the motion distortion effect varies between rotating and oscillating sensors, whereby oscillating sensors show a higher magnitude of the effect. Because of this higher magnitude of the motion distortion effect for oscillation scan patterns, we use a MEMS-based LiDAR sensor with an oscillating scan pattern in this work.

The sensor used in this study is a Blickfeld Cube 1. This LiDAR sensor is a MEMS-based scanning LiDAR sensor with a wavelength of 905 nm [12]. The functional principle of this ToF, scanning, oscillating sensor is discussed in the following section.

### 2.1. MEMS-Based LiDAR Sensors

A MEMS-based LiDAR sensor uses scanning mirrors constructed from silicon using photolithography to deflect the emitting beam pulses of a laser in a specific direction in the environment [12]. Because of the solid mechanical connection of the mirrors in the silicon structure, which vibrate in their resonant frequency, these sensors are classified as quasi-solid-state LiDARs [13].

The sensor in this work uses two 1D MEMS-based mirrors with orthogonal axes of motion to deflect the emitted laser beam pulses in defined azimuth and elevation angles [14].

Figure 1 shows a block diagram of the MEMS-based LiDAR used within this work. A 905 nm laser diode emits a laser pulse through a beam splitter. After this, the two MEMS-based mirrors deflect the laser pulse in a specific direction, with defined azimuth and elevation angles, in the FoV. In the environment, the laser pulse is partly reflected from an object’s surface back into the sensor’s optics and from the beam splitter to a solid-state detector, which converts the photons into an electrical voltage. After this, the electrical signal is processed to one point in the point cloud frame.

### 2.2. Sensor-Specific Scan Pattern

**Figure 3:**
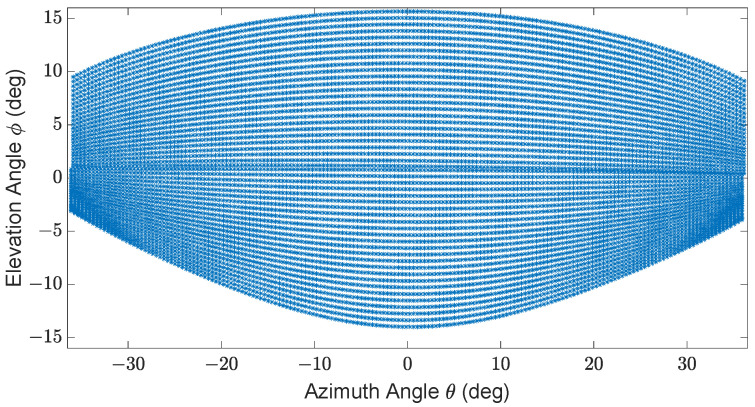
Exemplary scan pattern of Cube 1 with ±36${}^{\circ }$ horizontal and ±15${}^{\circ }$ vertical FoV, 50 scan lines, 0.4${}^{\circ }$ horizontal angle spacing, and a frame rate of 5.4 Hz [16].

To scan the entire FoV, a specific pattern is used. The sensor for this work allows the use of different scan patterns for a maximum FoV of ±36${}^{\circ }$ horizontal and ±15${}^{\circ }$ vertical, with a vertical resolution between 5 and 400 scan lines per frame and a horizontal resolution of 0.4${}^{\circ }$ to 1.0${}^{\circ }$. The frame rate of the sensor varies with the resolution [15]. Within the selected FoV, the sensor scans the environment with the chosen resolution. Figure 3 shows an exemplary scan pattern of the sensor.

### 2.3. Sensor-Specific Motion Distortion

The sensor used in this paper scans the FoV two times to generate one frame. The sensor scans the FoV from bottom to top (up scan) and then from top to bottom (down scan). The time duration between each scan point is 10.2 μs at a frame scan frequency of 5.4 Hz [16]. The distance *d* that the object moves from one point to another can be calculated from the relative velocity of the object *v* and the duration between two points Δ. For the sensor used,

(2) Δ=10.2 μs.

The distance that the object has traveled between *n* points can be calculated by

(3) d=n·Δ·v,

where *n* can be calculated from the point id of two points:

(4) $$n=id_{2}-id_{1}.$$

**Figure 4:**
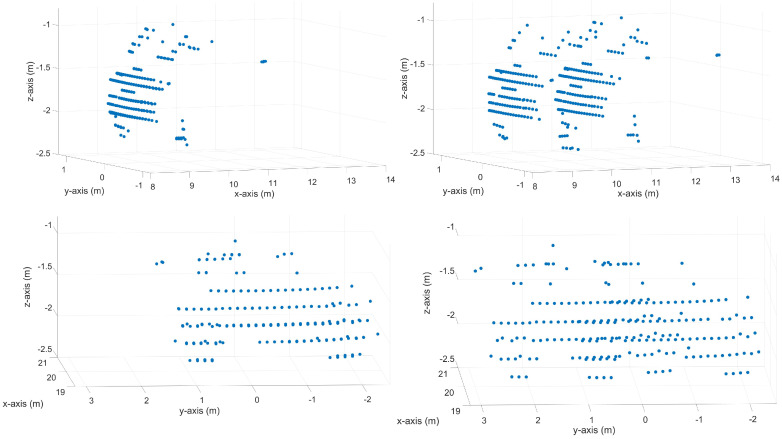
Synthetic point clouds of a vehicle. The top left shows a point cloud section of a vehicle with driving orientation in the x-direction with a relative velocity of 0.0 m s^−1^ (without a motion distortion effect). The top right shows a point cloud section of a vehicle moving in the x-direction with a motion distortion effect of 18.61 m s^−1^. The bottom left shows a point cloud section of a vehicle with driving orientation in the y-direction with a relative velocity of 0.0 m s^−1^ (without a motion distortion effect). The bottom right shows a point cloud section of a vehicle driving in the y-direction with a motion distortion effect with a relative velocity of 16.94 m s^−1^.

Figure 4 visualizes two measured point cloud sections, with and without the motion distortion effect. On the top, a point cloud section of a vehicle driving in the x-direction in the sensor coordinate system is shown, once with a relative velocity of 0.0 m s^−1^ (without the motion distortion effect) and once with a relative velocity of 18.61 m s^−1^ (with the motion distortion effect). The bottom of Figure 4 shows a point cloud section of a vehicle driving in the y-direction in the sensor coordinate system, once with a relative velocity of 0.0 m s^−1^ (without the motion distortion effect) and once with a relative velocity of 16.94 m s^−1^ (with the motion distortion effect). The position and orientation of the sensor coordinate system are shown in Figure 6.

The motion distortion effect will increase with an increasing relative velocity between the object and the sensor or a decreasing sensor frame rate. In addition, the degree of distortion depends on the position and extent of an object in the FoV.

Here, it should be noted that in the point clouds of the vehicles shown in Figure 2 and Figure 4, not only is a second shape of the trunk created by the time difference between the up (Figure 2 blue) and down scan (Figure 2 red), but the objects are also distorted in themselves. Thereby, it is likely generating a false positive object recognition and negatively affects the object detection. However, this distortion is not visible due to the plot’s scaling and the small point cloud section of the objects from the entire LiDAR frame. A false positive object occurs here because the VoI does not cover the entire FoV or the object’s position is not at the top of the FoV where the up and down frames meet.

## 3. State of the Art

Different aspects of the motion distortion effect from scanning LiDAR sensors are well described in the literature. Ballard and Vacherand [17] show a correlation between the frame rate of the sensor and the velocity and trajectory of the moving object. Ono et al. [18], Ji et al. [19], and Gröll and Kapp [20] describe the problem arising from the motion distortion effect using maps of scanning LiDAR sensors. In [16], the authors introduce a simulation method to generate high-fidelity synthetic point clouds, including the motion distortion effect. Several papers show different means of correcting for the motion distortion in a point cloud. Renzler et al. [21] and Muro et al. [22] propose compensation methods for the motion distortion effect caused by a moving ego vehicle. Methods to compensate for the motion distortion effect caused by a moving ego vehicle are shown by Renzler et al. [21] using odometry, Muro et al. [22] using sensor data from an inertial measurement unit (IMU), and Merriaux et al. [23] using CAN-bus data. To correct the motion distortion of moving OoIs, Yang et al. [11] fuse the sensor data of the LiDAR sensor with an RGB camera and use tracking to estimate the velocity of an OoI. To calculate the velocity of OoIs in LiDAR point clouds, different tracking methods are currently used. For single-object tracking, Siamese tracking is often used. Here, the OoI is searched for in sequential frames in the surroundings of the object detected in the previous frames [24]. For LiDAR sensors, the object’s shape in the point cloud is considered in this approach, as in [25,26]. With multi-object tracking, several different objects can be tracked. These methods track the bounding boxes based on the shape and movement of the objects over several frames, as in [27,28]. Based on the object tracking, the object’s velocity can then be determined based on multiple frames, as described in [28]. In [29], Gu et al. show improved tracking and segmentation when using the velocity information from an FMCW LiDAR point cloud. Most state-of-the-art research papers show the consequences of the motion distortion effect or focus on correcting the point cloud distortion. Mainly, the papers deal with the distortion by using sensor data fusion to correct the distortion from the ego vehicle movement. Current research satisfies the velocity estimation via the sensor data fusion of an OoI, which is the baseline for the correction of the distortion of this object in the point cloud. To be able to calculate the velocity of the OoI, tracking over multiple frames is used. The extraction of an OoI’s velocity directly from the motion distortion in a point cloud still needs to be investigated.

Therefore, in this work, we introduce a neural network-based approach to estimate the velocity and direction of motion of an OoI directly from a single point cloud, called VeloPoints. To the best of the authors’ knowledge, this is the first work estimating the velocity of an OoI object from only one single LiDAR point cloud without any sensor data fusion or tracking. The extracted velocity information (4D-LiDAR) helps in motion prediction and object tracking, leading to more reliable data due to more redundancy for sensor data fusion. The associated corrected point clouds are promising for better object recognition and detection [30,31].

## 4. Generation of Labeled Dataset

**Figure 5:**
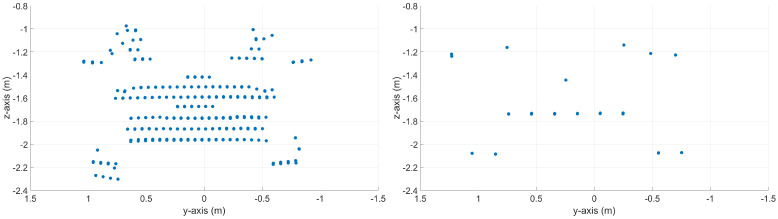
Comparison of point cloud density of a vehicle, on the left side at a distance of 8.0 m and on the right side at 28.0 m.

Point clouds from a scanning LiDAR sensor offer a lower resolution than state-of-the-art camera-image-based methods. Due to the correlation between the distance of an object from the sensor and the point density on the object, the number of measured points decreases with increasing distance [32]. The decrease in the point density of an object with increasing distance is shown in Figure 5. Due to the lower resolution of scanning LiDAR sensors compared to, e.g., state-of-the-art camera-image-based measurement methods, an analytical approach to determining the velocity from motion distortion is difficult to implement. To calculate the velocity, a change in distance would have to be derived from a known time duration. One must uniquely identify two identical measuring points on the distorted object to calculate a distance change. However, this is normally not possible due to the spatial resolution of the LiDAR point cloud. Nevertheless, the distortion of the point cloud in the area of a moving object represents clear patterns. Since machine learning methods are particularly suitable for the extraction and learning of patterns, in this study, we use machine learning methods—more precisely, artificial neural networks.

A labeled dataset is required to train and validate artificial neural networks using supervised learning. A simulation was used since creating such a dataset on a sufficient scale in the real world is costly. For this purpose, the simulation software CarMaker from the IPG Automotive GmbH was combined with the high-fidelity sensor models of the Cube 1 scanning LiDAR sensor presented in [16], which considers sensor-specific imperfections. To reduce the computation time of the simulation, the motion distortion effect was modeled on the simulated point cloud using the analytical approach described in [16]. Therefore, a point cloud was simulated with a single-shot simulation (like a flash LiDAR sensor) and the scanning effects were applied afterward on the point cloud.

**Figure 6:**
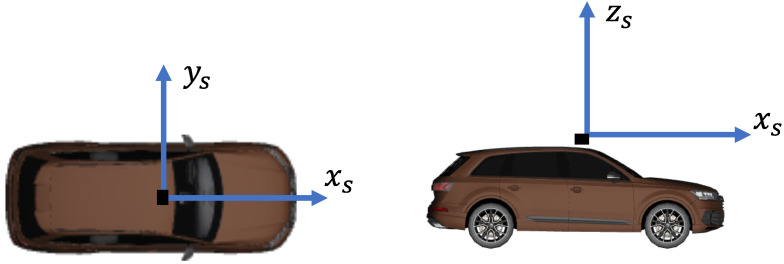
Position of the sensor and the sensor coordinate system alignment on the virtual vehicle.

For the simulation of a dataset, a virtual vehicle was equipped with a virtual LiDAR sensor on the roof in the middle of the car at a height of 2.3 m, which corresponds to the height of the sensor on an Audi Q7 with a sensor roof mount. Figure 6 shows the position of the sensor on the virtual vehicle and the orientation of the sensor coordinate system.

The virtual LiDAR sensor was configured with a sensor-specific scan pattern with the parameters shown in Table 1. Within the parameters possible in the LiDAR sensor used in this work, the chosen scan pattern exhibited a sufficiently large FoV and a sufficiently accurate resolution for the use case of an ADAS application.

**Figure 7:**
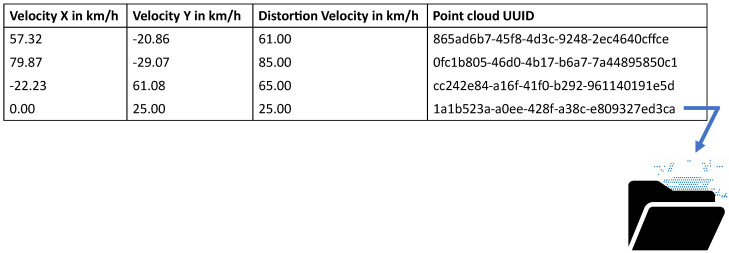
Structure of the dataset used for the training, optimization, and testing of the neural network.

The neural network should be able to estimate an object’s velocity and direction of motion from the generated synthetic point clouds. Therefore, these parameters must also emerge from the dataset for supervised learning. The resulting structure of the dataset consists of the resultant velocity in km h^−1^, the coordinate velocity of the object in $x_{s}$- and $y_{s}$-coordination in km h^−1^ in the sensor coordinate system, and the Universally Unique Identifier (UUID) of the point cloud. The structure of the dataset is shown in Figure 7.

The coordinates $x_{s}$, $y_{s}$, and $z_{s}$ of the measured points in the point cloud of the LiDAR sensor are not stored directly in the data frame of the dataset due to efficiency reasons, but are stored externally as binary files provided with an inimitable UUID consisting of the Coordinated Universal Time, the clock sequence, and the IEEE 802 MAC address, as described in [33]. In the dataset, the point clouds are referenced using only their UUIDs. By using binary files instead of point clouds in CSV format, the required memory capacity for the dataset can be reduced by 70.4%.

**Figure 8:**
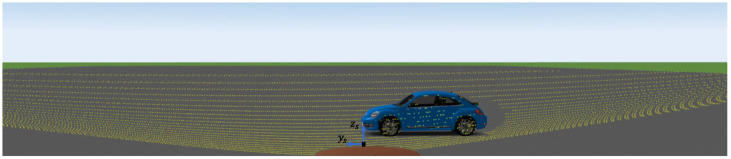
Example visualization of a simulation scenario. The VoI is driving in $y_{s}$-direction in front of the ego vehicle.

For the simulation of the point clouds, the virtual vehicle described above was positioned in the simulation environment; see Figure 8. Afterward, different simulation trajectories were driven by a VoI in the FoV of the virtual LiDAR sensor. The different trajectories were varied in distance between 2.0 m and 50.0 m from the ego vehicle, with different offsets in the $x_{s}$-direction and $y_{s}$-direction, based on the following and crossing scenarios from the field of ADAS, with different angles between ±30.0${}^{\circ }$ rotated around the $z_{s}$-axis. The object is still recognizable in the point cloud in these value ranges, and sufficient scenario variation is still achieved. The ego vehicle stands still to represent only the influence of the object’s velocity. However, this circumstance should not cause any difficulties for future applications concerning velocity estimation based on the motion distortion effect, since the additional distortion of the point cloud due to the self-motion of the sensor can be compensated for based on the data of the IMU of the sensor or the vehicle. Thus, the distortion resulting from the self-motion can be compensated for.

In this setup, the virtual sensor recorded 12,684 different positions of the VoI. The velocities were within a vehicle-typical range between 5.55 m s^−1^ and 33.33 m s^−1^ (20.0– 120.0 km h^−1^). In total, 127,249 different point clouds with different VoI positions and velocities were simulated. Dividing the dataset into velocity ranges of 2.77 m s^−1^ (10.0 km h^−1^), the number of frames in each velocity range showed a mean absolute deviation of 2.5%. In the following, the data generated with this method will be used to train, optimize, and test an artificial neural network, as described in the following sections.

## 5. VeloPoints Network

**Figure 9:**
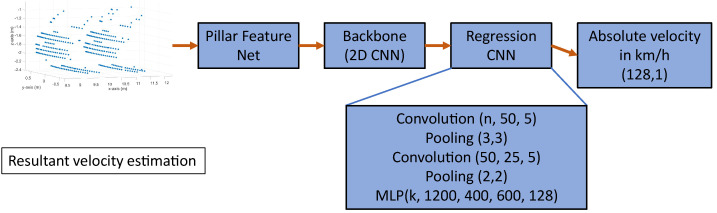
Structure of the VeloPoints network for the resultant velocity estimation with a multilayer perceptron (MLP) as a multi-regression artificial neural network.

**Figure 10:**
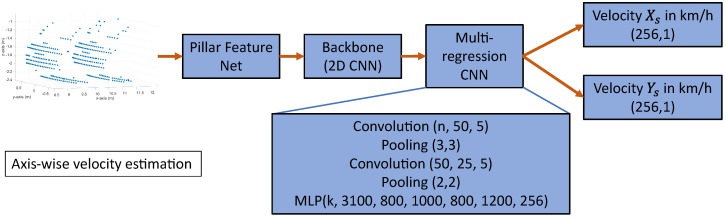
Structure of the VeloPoints network for the axis-wise velocity estimation with a multilayer perceptron (MLP) as a multi-regression artificial neural network.

To estimate the velocities and direction of motion of an object from a single point cloud of a scanning LiDAR sensor, we developed a neuronal network called VeloPoints. VeloPoints is an artificial neural network for the estimation of the velocities of an object in a point cloud based on the motion distortion effect of a scanning LiDAR sensor. The network’s input is a 3D point cloud of a LiDAR sensor consisting of points with the coordinates $x_{s}$, $y_{s}$, and $z_{s}$. To apply a 2D convolution architecture commonly used in image processing for feature extraction, the VeloPoints network uses the feature encoder network of Lang et al. [32]. The feature encoder network converts the sparse 3D point cloud, discretizing the $x_{s}$-$y_{s}$ plane to create pillars, which are then converted into a 2D sparse pseudo image [34]. Subsequently, the 2D convolution backbone from [32,34] obtains a high-level representation from the 2D sparse pseudo image. Afterward, a custom multi-regression convolutional neural network (CNN) estimates the velocity from the extracted features. We developed two different derivatives of the VeloPoints network to cover different future use cases. The first variant estimates the resultant velocity of the OoI from the distortion of the object in the point cloud, and the second variant estimates the velocity axis-wise along the $x_{s}$- and $y_{s}$-axis in the sensor coordinate system. By using the network with an axis-wise estimation, the resultant velocity of the object and its direction of motion can be calculated. However, the network with the estimation of the resultant velocity has a simpler structure and a higher prediction speed. The structures of the two VeloPoints network variants are shown in the following figures. Figure 9 shows the structure of the network for the resultant velocity estimation, and Figure 10 shows the structure for the axis-wise velocity estimation.

**Figure 11:**
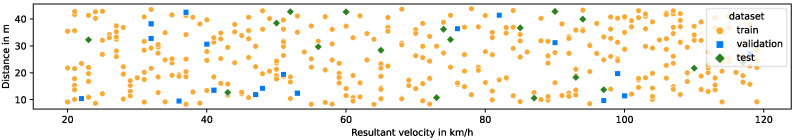
Distribution of a subset of data points over distance for an exemplary simulated scenario, in which the VoI was driving in the distance from 8.3 m s^−1^ to 44.4 m in the FoV of the sensor, and the split into training, validation, and test data.

The synthetic dataset described above was divided into training, validation, and test datasets, as shown in the following Table 2, to train, validate, and test the neural networks. Figure 11 shows the distribution of a subset of data points over distance for an exemplary simulated scenario, in which the VoI was driving in the distance from 8.3 m to 44.4 m in the FoV of the sensor, as well as the division into training, validation, and test data.

The training dataset was used to fit the weights in the artificial neural network. In parallel, after each epoch, the performance of the current neural network was evaluated using the model’s unknown validation data while still maintaining the training. The most promising models for both variants were finally tested with data from the test dataset, which was unknown to them until that point.

In conjunction with the training, the following artificial neural network hyperparameters were optimized to achieve the best possible result:
Number of convolutionsLoss functionNumber of layers in the MLPInitial learning rateNumber of neurons per layerMax momentumNetwork architectureBatch sizeActivation functionEpochs

## 6. Results

During the development of the two neural network variants of the VeloPoints method, results were obtained at different stages. The results of the hyperparameter optimization, the estimation of the object velocity of the two models, and the estimation accuracy over the whole velocity range are considered in more detail below.

### 6.1. Hyperparameter Optimization

The trained models of VeloPoints were validated in two stages. During the training and optimization, the generated models were tested with the help of the validation dataset. With this, the output value of the loss function, as well as the mean absolute error (MAE) and the root mean squared error (RMSE) from the velocity estimations of the models and the corresponding labels in the dataset, were evaluated on both the training and validation datasets. In this way, not only the training state of the model was monitored in terms of overfitting or underfitting, but also the hyperparameters were optimized. The most promising models for the optimization of the hyperparameters with the lowest RMSE during this first validation were then validated again with the previously unknown test dataset. Again, the MAE and RMSE of the test dataset were considered. The hyperparameters of the best models, both for the resultant velocity estimation variant and the axis-wise velocity estimation variant, are shown in Table 3.

The obtained results of the optimized models for each VeloPoints variant, with the hyperparameters shown in Table 3, are discussed in the following sections.

### 6.2. Absolute Velocity Model

**Figure 12:**
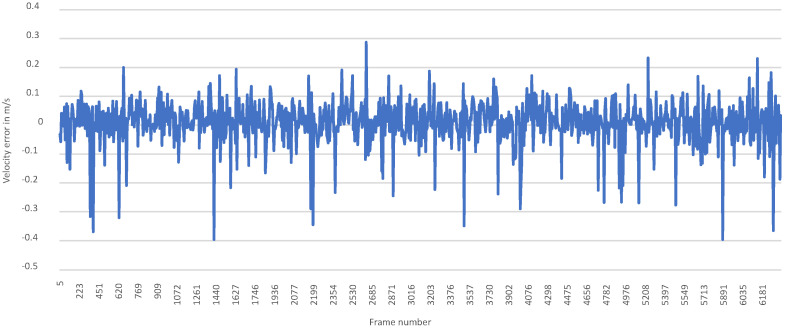
Error for each frame in the test dataset between the resultant velocity estimation by VeloPoints and the corresponding labels in m s^−1^.

Figure 12 shows the error between the resultant velocity estimated by VeloPoints and the labels in the test dataset in m s^−1^.

**Figure 13:**
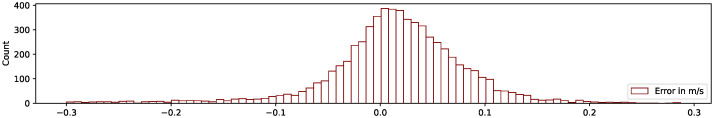
Histogram of the estimation error of the model with resultant velocity estimation in m s^−1^.

The error between the resultant velocity estimation of the model and the distortion velocity label in the dataset, calculated from all the data points in the test dataset, is normally distributed, as shown in the following histogram in Figure 13.

The model with the resultant velocity estimation and optimized parameters achieves an RMSE of 0.0815 m s^−1^ and an MAE of 0.0526 m s^−1^. The 95.4% confidence interval, meaning two sigma for the estimation error, is [0.0138 m s^−1^, 0.0170 m s^−1^]. Of the estimated velocities from the model, 98.8% have an error of less than 0.27 m s^−1^ (1 km h^−1^).

### 6.3. Axis-Wise Velocity Model

**Figure 14:**
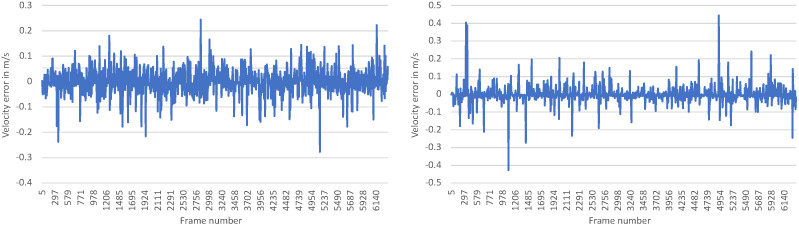
Error for each frame in the test dataset between the velocity estimation by VeloPoints and the corresponding labels in m s^−1^ for the x-axis on the left and y-axis on the right.

Figure 14 shows the error between the VeloPoints estimation and the test dataset labels for the x- and y-axis in the sensor coordinate system.

**Figure 15:**
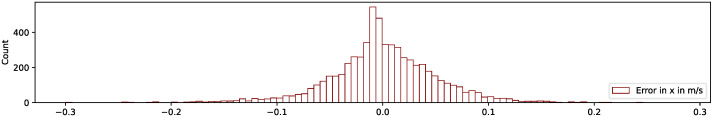
Histogram of the estimation error in x-direction of the model with axis-wise velocity estimation in the sensor coordinate system in m s^−1^.

The error between the axis-wise velocity estimations of the model and the velocity in the x- and y-direction labels in the dataset, calculated from all the data points in the test dataset, is normally distributed. This normal distribution is shown by the following histogram in Figure 15 as an example based on estimation errors in the x-direction of the sensor coordinate system.

The model with optimized parameters achieves a combined RMSE of 0.1187 m s^−1^ and a combined MAE of 0.038 m s^−1^ for both axes. The 95.4% confidence interval, meaning two sigma, for the estimation error, for example, in the x-direction is (−0.0008 m s^−1^, 0.0017 m s^−1^). Of the estimated velocities from the model, 98.9% have an error in the x- and *y*-directions of less than 0.27 m s^−1^ (1.0 km h^−1^). Thus, both models accurately estimate the axis-wise velocities in the x- and y-directions or the resultant velocity. By outputting the velocity in the x- and y-directions, information is not only available about the axis-related velocity of the object but also about the resultant velocity and the direction of motion of the object. In contrast to the axis-wise estimation model, the model for the estimation of the resultant velocity has a simpler network structure. Thus, the network for resultant velocity estimation is less computationally expensive and has a shorter prediction time than the network for axis-wise velocity estimation.

### 6.4. Analysis of the Influences on the Estimation

To consider different influences on the velocity estimation, the dependency of the relative velocity between the VoI and the sensor and the shape of the VoI on the estimates are considered in this section.

#### 6.4.1. Velocity Influence on the Estimation

**Figure 16:**
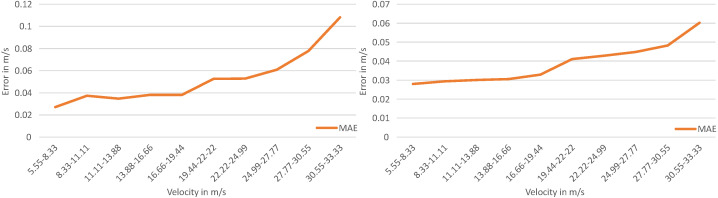
Effect of the velocity on the estimation accuracy. The diagram on the left shows the MAE in m s^−1^ for the resultant velocity estimation model. The diagram on the right shows the MAE in m s^−1^ for the axis-wise velocity estimation model.

To determine the effect of different velocities on the prediction accuracy, all LiDAR frames in the dataset are divided based on the velocity of the OoI in 2.77 m s^−1^ (10.0 km h^−1^) intervals. Therefore, the test data are divided into intervals, and the MAEs are calculated for each interval. Figure 16 shows the results for the model with axis-wise velocity estimation (left) and the model with resultant velocity estimation (right).

Figure 16 shows that the MAE for the velocity estimation for both the model with axis-wise velocity estimation and the model with resultant velocity estimation indicates a larger deviation in the velocity estimation with the increasing velocity of the OoI.

**Figure 17:**
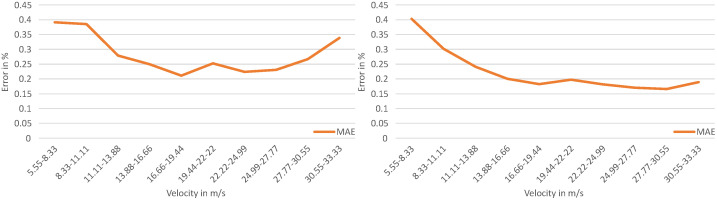
Percentage deviation of the estimated velocity based on the mean of the velocity intervals. The diagram on the left shows the percentage deviation for the resultant velocity estimation model. The diagram on the right shows the percentage for the axis-wise velocity estimation model.

Comparing the percentage deviation of the MAE from the mean value of the individual velocity intervals in Figure 17, no clear dependence between the velocity of the OoI and the velocity estimate can be seen relative to the mean value of each interval for both the model with axis-wise velocity estimation and the model with resultant velocity estimation. Thus, an increasing absolute MAE occurs as the velocity of the OoI increases. However, the relative MAE does not show a clear dependence between the deviation and the velocity. For this reason, the deviation should be considered on an application-specific basis for datasets with large or small velocities. With a maximum deviation of 0.40% for the model with axis-wise velocity estimation and 0.39% for the model with resultant velocity estimation to the mean value of the corresponding interval, both models show excellent estimation performance.

#### 6.4.2. Shape Influence on the Estimation

To determine the influence of the OoI’s shape on the velocity estimation, the dataset is divided into two scenarios: the OoI is moving only in the x-direction, and the OoI is moving only in the y-direction in the sensor coordinate system. For both sub-datasets, the velocity estimation results obtained from VeloPoints are compared. The estimate’s accuracy for the scenario with the OoI driving in the x-direction is only 0.2001 percentage points higher than for scenarios with the OoI driving in the y-direction. This means that the VeloPoints velocity estimation currently shows no dependency on the shape of the OoI.

## 7. Conclusions

In this paper, we present a method of estimating the velocity from a point cloud of a scanning LiDAR sensor based on the motion distortion effect. For this purpose, a synthetic dataset with the motion distortion effect for the training of neural networks was created with the help of validated high-fidelity sensor models, as shown in [14,16]. Subsequently, the generated dataset was used to train two VeloPoints neural network variants that predict either an object’s absolute or axis-wise velocity of a point cloud. Both model variants show outstanding results on test data. To the best of the authors’ knowledge, VeloPoints is currently the only method available to estimate the velocity of an object in a point cloud with only one LiDAR sensor and a single frame, without sensor data fusion or object tracking. Based on the results obtained, the information extracted by the VeloPoints network can be used for motion prediction. Using VeloPoint, object detection and simultaneous localization and mapping can be improved by compensating for the distortion of the point clouds based on the known velocity and duration between the scan points. This allows the characteristic shapes of objects to be reconstructed. In addition, the velocity information extracted from the VeloPoints network can be used to optimize object tracking. From the first frame in which an object is detected for object tracking, the velocity information can be provided from VeloPoints as an input parameter for the object tracking method.

In addition, the speed information extracted from the VeloPoints network can flow into the sensor data fusion module and make it more reliable, as different speed measurement methods can be fused competitively and the information is available more quickly, as only one frame is required for the velocity estimation. This optimized sensor data fusion can serve as a superior decision-making basis for autonomous systems.

## 8. Outlook

Based on the method of velocity estimation from a single point cloud of a scanning LiDAR sensor using a neural network developed in this work, the influence of real data on the two different derivatives of the VeloPoints network will be addressed in the next step. Based on the measured data, the effects of further scenario-dependent influences on the velocity estimation, such as the object’s distance, the position of the OoI in the FoV of the sensor, and different types of objects, as well as various network designs, will be considered. In addition, it is relevant to consider the existing method of compensating for the motion distortion effect and its use in sensor data fusion. Furthermore, extending the dataset used in this paper can improve the network’s performance, and an analysis of the influence of different effects—for example, an efficient mixture of real and simulated data in the dataset—needs to be performed.

## Figures and Tables

**Table 1:** Parameters of the scan pattern used to generate the dataset.

Scan Pattern Parameter	Value
FoV horizontal	72.0 ${}^{\circ }$
FoV vertical	30.0 ${}^{\circ }$
Number of scan lines	100
Horizontal spacing	0.4 ${}^{\circ }$
Frame rate	5.4 Hz

**Table 2:** Number of data points in the datasets.

Dataset	Number of Data Points
Train	114,523
Validation	6363
Test	6363

**Table 3:** Hyperparameters of the best models for the two estimation variants.

Hyperparameter	Resultant Velocity Model	Axis-Wise Velocity Model
Number of convolutions	2	2
Number of layers in the MLP	5	6
Number of neurons per layer	(k, 1200, 400, 600, 128, 1)	(k, 3100, 800, 1000, 800, 1200, 256, 2)
Network architecture	Convolution + MLP	Convolution + MLP + 2x TLP
Activation function hidden layer	Relu/Linear	Relu/Linear
Activation function output layer	Relu/Linear	Relu/Linear
Loss function	Huber Loss [35]	Huber Loss [35]
Initial learning rate	0.0000125	0.0000125
Max momentum	0.99	0.99
Batch size	16	16
Epochs	183	179

## Data Availability

Data are contained within the article.
